# Identifying anticipated barriers to help-seeking to promote earlier diagnosis of cancer in Great Britain

**DOI:** 10.1016/j.puhe.2016.08.012

**Published:** 2016-12

**Authors:** J. Moffat, R. Hinchliffe, L. Ironmonger, K. Osborne

**Affiliations:** Cancer Research UK, Angel Building, 407 St John Street, London EC1V 4AD, UK

## Abstract

• On average, people endorsed three barriers that would put them off seeing a GP.
• Women, younger people and more deprived groups were more likely to endorse barriers.
• Service-related barriers endorsed more than barriers related to GP–patient relations.
• Endorsement of not wanting to be seen making a fuss reveals more social element.
• The results suggest that there may be additional barriers influencing patient behaviour.

## Introduction

Understanding the public's awareness of, and response to, symptoms which could be cancer has been an important element of the work to promote earlier cancer diagnosis.^1^, ^2^ This has included identifying and assessing anticipated barriers to help-seeking, with previous research identifying the most frequently endorsed barriers, and variations in endorsement of barriers, by different groups.^3^, ^4^ Difficulty making an appointment, worry about wasting the doctor's time, and worry about what would be found were some of the most commonly endorsed barriers in a sample of more than 2,000 British people, with lower socio-economic groups more likely to report ‘emotional’ barriers and higher socio-economic groups more likely to report ‘practical’ barriers.^5^ International comparisons of cancer awareness and beliefs have also been made and have reported greater endorsement of barriers to help-seeking in UK adults compared to adults from Sweden, Norway, Denmark, Australia or Canada, particularly for being worried about wasting the doctor's time.^6^ Given that the barriers healthy people endorse may be different to the ones which influence their behaviour in the event of symptoms, studies have also sought to assess which barriers seem important for actual behaviour (see Forbes et al.^7^).

Understanding the potential barriers to help-seeking in the event of symptoms is crucial to inform efforts to mitigate those barriers. Recognition that some people are deterred from seeking help because of a worry about wasting the GP's time, for example, is one of the reasons why GPs feature heavily in the creative materials for most of England's ‘Be Clear on Cancer’ awareness campaigns.^8^ But the response options used to gauge barriers to help-seeking within the Cancer’ Awareness Measure (CAM) to date are fairly broad and have not been revisited since the late 2000s. There is an opportunity, as well as a need, to further explore these barriers and ensure we are capturing those most salient to our population.

## Materials and methods

We used a modified version of the CAM to collect data on barriers towards visiting the doctor. The CAM is a validated set of questions designed to reliably assess awareness of cancer.^9^ Every 2 years, since 2008, Cancer Research UK (CRUK) has run the CAM via the Opinions and Lifestyle Survey (OLS), an omnibus survey run by the Office for National Statistics (ONS) using a representative sample of the population of Great Britain. The OLS survey recruits using random probability sampling (see Appendix A) and is conducted in respondents' homes via face-to-face, computer-assisted interviews. Adults aged 16 years and over are eligible to participate in the survey.

Prior to the 2014 survey, an expert group was established to review items within the CAM and make recommendations for changes and additions. Potential items were then refined by CRUK and piloted using their online audience research panel, followed by further refinement prior to inclusion. The questions and response options for anticipated barriers to help-seeking items were enhanced as part of this process and validation of these is pending.

The data used in this analysis were collected in October and November 2014 and are available in the UK Data Archive. Multivariable logistic regression was used to calculate adjusted odds ratios for endorsement of barriers to seeing a GP by demographic characteristics and a self-reported measure of health (see Appendix A). Models were initially developed using backward stepwise regression with further exploration including interaction terms. Analyses were carried out using STATA 13.^10^

## Results

### Participants

The response rate was 54% (1,986/3,677); 34% refused, 1% had unknown eligibility and 10% could not be contacted after three attempts. The overall sample size was 1,986 (55%, *n* = 1,092 women). The mean age was 53 years (SD = 18.7), and most participants were in a relationship (53.5%) and white (90.9%) (Table 1).

### Overall

Participants were considered to have endorsed a barrier if they responded ‘strongly agree’ or ‘agree’ to questions about barriers to help-seeking (see Appendix A for full item wording). On average, people endorsed three barriers that would put them off seeing a GP (3.02, SD = 2.66). The most frequently endorsed barriers to seeing a GP were service related; difficulty getting an appointment with a particular doctor (41.8%) and difficulty getting an appointment at a convenient time (41.5%). The next most frequently endorsed barriers have both service and emotional elements; dislike of having to speak to the GP receptionist about symptoms (39.5%) and not wanting to be seen as someone who makes a fuss (34.8%). Barriers that were least often endorsed were finding the GP difficult to talk to (7.3%), not feeling confident talking about their symptoms with the GP (8.6%) and finding it embarrassing talking to the GP about their symptoms (9.0%).

### Variation by demographic groups

In adjusted analyses (Table 1), there were significant differences by age for most of the barriers (10 of 14). In general, those aged 16–54 years were more likely to endorse the barrier than those aged 55–64 years, while those aged 65–74 years and 75+ years were less likely than those aged 55–64 years.

There were significant differences among men and women for 10 of the barriers. In all of these instances, men were less likely to endorse the barrier than women. For example, men were less likely to agree that they found it difficult to get an appointment with a particular doctor (OR: 0.63 [95% CI: 0.52–0.76]) or that they had had a bad experience at the doctor's in the past (OR: 0.68 [95% CI: 0.53–0.88]).

With regards to occupation, there were significant differences in six barriers, with respondents in non-managerial occupations, full-time study or not in work almost always more likely to endorse the barrier than those in managerial occupations. The only exception to this was endorsing being ‘too busy to make time to see the doctor’, with both full-time students and those not in work less likely to endorse this barrier (full-time students OR: 0.36 [95% CI: 0.16–0.82] and those not in work OR: 0.29 [95% CI: 0.17–0.50]).

There were only two significant differences among white and non-white groups, with non-white respondents being less likely to endorse not wanting to be seen as somebody who makes a fuss (OR: 0.64 [95% CI: 0.45–0.92]) and dislike of having to talk to the GP receptionist about their symptoms (OR: 0.64 [95% CI: 0.45–0.91]).

## Discussion

Identifying and assessing endorsement of anticipated barriers to help-seeking is useful for informing approaches to mitigate their impact and foster timely presentation to health services. Previous research has found endorsement of barriers to be higher in the UK than in countries with comparable health systems and has called for more research in this area.^6^ In response to this, we included additional response options within the barriers item of the CAM, which have provided new insight on factors that may deter people from seeing their GP at the earliest opportunity.

The high endorsement of service-related barriers chimes with previous work using the CAM, including the first nationally representative survey using the measure which reported the most commonly endorsed barrier to be difficulty making an appointment.^5^ Furthermore, we have been able to explore the service-related barriers in more detail through the new response options, which establish difficulty in getting an appointment with a particular doctor, difficulty in getting an appointment at a convenient time and dislike of having to speak to the GP receptionist about symptoms, as the most frequently endorsed anticipated barriers to help-seeking. It is not clear how much of this is perceived vs actual difficulty, but triangulation with data from, for example, the GP patient survey^11^ could provide useful insight here in future.

Interestingly, the more individual/GP-specific barriers, such as finding the GP difficult to talk to, not feeling confident talking to the GP about symptoms, and feeling embarrassed talking to the GP about symptoms, were the least frequently endorsed, suggesting that the wider service and structures in which GPs operate are a more significant barrier to help-seeking than factors associated with individual GP–patient relationships.

The original CAM included an item on ‘worry about wasting the doctor's time’, which has received high endorsement in previous research.^5^, ^6^ In the revision to the barriers items, we also included the new response option of not wanting to be seen as someone who makes a fuss. The ‘fuss’ item was endorsed more frequently than ‘worry about wasting the doctor's time’ (34.8% vs 19.5%). While the two items are similar, the ‘fuss’ barrier would seem to be tapping into a more socially driven concern wherein fears about how the individual is going to be perceived by others are central (as is explicit in the phrasing of the response option itself). Conversely, the factors involved in ‘worry about wasting the doctor's time’ might be more closely related to concerns about appropriate use of health services, which could also have implications for identity and how one is perceived by others, but these are less central. Indeed, since the response options were modified, a qualitative interview study by Whitaker et al.^2^ of individuals reporting recent experience of a cancer alarm symptom found that narratives relating to worry about wasting the doctor's time seemed to be more about self-identity than a primary intention to ration the use of health services.

However, because the fuss item is a new addition, it is not possible to know with certainty whether there have been real, meaningful shifts in endorsement of worry about wasting the doctor's time. It may be that the ‘fuss’ item is better tapping into the issues patients are grappling with which has led to a reduction in endorsement of the original item, or perhaps it is a broader concept which includes an array of more specific worries, including worry about wasting the doctor's time.

Regardless, the relatively high endorsement of the ‘fuss’ item suggests that it would be pertinent to further investigate its import and look at ways to address it, should it be considered to be significant for help-seeking behaviour. It has been suggested that it may be difficult to tackle barriers concerned with the perception of others^2^ and attitudes to GPs^12^ via public awareness campaigns. But national awareness activity such as England's ‘Be Clear on Cancer’ programme has demonstrated success in bringing people experiencing campaign-related symptoms forward to their GP.^8^ It is not unreasonable to assume that in doing so, the campaigns have helped to address barriers to help-seeking, and indeed the decision to make real-life GPs a prominent feature of most of the campaigns was to help reinforce help-seeking behaviour from a GP voice. It may be possible to extend this messaging to more explicitly address the ‘fuss’ element and, if combined with strong and reinforcing GP level interactions and other local activity, it could go a long way to breaking this barrier down.

The analysis showed that women, the youngest age groups and those with lower socio-economic status (as measured here by occupation) endorsed the most barriers to help-seeking and this is broadly consistent with previous work (see Niksic et al.^3^). Further understanding of these demographic differences, and their associations with actual help-seeking behaviour, would be useful. This would enable prioritization of efforts to address these inequalities, and inform the nature and content of those efforts to facilitate the tailoring of activities and optimize the translation of evidence into policy and practice.

### Limitations

Whilst the sampling for the OLS is of a good standard, results are based on a self-selecting sample who are asked to respond to a hypothetical scenario and select from specific response options. It is not possible to discern from this work the extent to which these factors would actively deter someone from seeking help in the real-life event of experiencing a symptom, but the results do provide a useful foundation for further work.

### Conclusions

These findings add to the evidence on the barriers that may influence the public's decisions around help-seeking in the event of a symptom and suggest that there may be different or broader barriers, in addition to the ones previously considered, which may be of particular significance for influencing the timeliness of help-seeking behaviour. Further exploration of these ‘new’ barriers and understanding of the extent to which the barriers are associated with actual help-seeking behaviour would be helpful to then inform the development of policy or other interventions to mitigate their impact. Similarly, the confirmation of the sociodemographic inequalities in the extent to which these barriers are experienced, and differences in which particular barriers are most often endorsed, helps us to specifically target future activity and work to lessen the barriers for everyone.

## Author statements

### Acknowledgements

We thank Dr Emily Power for leading on the process for reviewing and updating the CAM items and reviewing a previous version of this article. We also thank the late Professor Jane Wardle who was integral to the development of the original CAM and, in the years following, offered much valuable input and advice to the team at Cancer Research UK.

### Ethical approval

The authors confirm that they have observed appropriate ethical guidelines and legislation in conducting the study described in this paper.

### Funding

The work was initiated and funded by Cancer Research UK.

### Competing interests

The authors declare no conflict of interest.

## Figures and Tables

**Table 1:** Endorsement of barriers to help-seeking by demographic groups.

Q: Which of the following might put you off going to the doctor?		Total	Gender		Age in years						Occupation (socio-economic status)					Ethnicity		Relationship status		Long-standing illness/disability/infirmary	
			Women	Men	16–24	25–44	45–54	55–64	65–74	75+	Managerial	Intermediate	Routine	Full-time students	Unclassified^a^	White	Non-white	No partner	Partnered	No	Yes
a) I find it embarrassing talking to the doctor about my symptoms	% (*n*)	9.0 (174)	10.3 (110)	7.3 (64)	20.4 (29)	10.7 (59)	8.4 (26)	8.3 (28)	4.8 (16)	6.0 (16)	7.8 (41)	8.4 (29)	10.1 (44)	20.8 (15)	7.9 (45)	9.1 (161)	7.9 (13)	9.9 (89)	8.2 (85)	8.6 (94)	9.4 (80)
	OR (95% CI)^b^	–	1.00	**0.71 (0.51–0.99)**	**3.26 (1.83–5.81)**	1.44 (0.89–2.33)	1.06 (0.61–1.86)	1.00	0.54 (0.29–1.03)	0.64 (0.34–1.22)	–	–	–	–	–	–	–	–	–	1.00	**1.51 (1.08–2.12)**
b) I would be worried about wasting the doctor's time	% (*n*)	19.5 (380)	21.1 (226)	17.6 (154)	26.8 (38)	18.8 (104)	20.1 (62)	19.4 (65)	18.5 (62)	18.2 (49)	16.1 (84)	21.5 (74)	20.5 (89)	27.8 (20)	19.8 (113)	20.0 (355)	15.2 (25)	21.1 (190)	18.2 (190)	18.7 (204)	20.8 (176)
	OR (95% CI)	–	1.00	**0.80 (0.63–1.00)**	–	–	–	–	–	–	–	–	–	–	–	–	–	–	–	–	–
c) My doctor is difficult to talk to	% (*n*)	7.3 (141)	8.3 (89)	5.9 (52)	15.5 (22)	7.6 (42)	9.4 (29)	6.0 (20)	4.5 (15)	4.8 (13)	6.5 (34)	7.3 (25)	8.5 (37)	9.7 (7)	6.7 (38)	7.2 (128)	7.9 (13)	8.2 (74)	6.4 (67)	7.1 (78)	7.4 (63)
	OR (95% CI)	–	1.00	**0.70 (0.49–1.00)**	**2.95 (1.55–5.60)**	1.29 (0.75–2.25)	1.66 (0.92–3.00)	1.00	0.77 (0.39–1.53)	0.80 (0.39–1.64)	–	–	–	–	–	–	–	–	–	–	–
d) I find it difficult to get an appointment with a particular doctor	% (*n*)	41.8 (813)	47.0 (502)	35.5 (311)	51.4 (73)	45.2 (250)	42.1 (130)	40.2 (135)	36.6 (123)	37.9 (102)	37.1 (194)	47.8 (165)	46.3 (201)	38.9 (28)	39.4 (225)	42.1 (749)	38.2 (63)	41.5 (374)	42.1 (439)	40.0 (437)	44.2 (375)
	OR (95% CI)	–	1.00	**0.63 (0.52–0.76)**	**2.14 (1.35–3.38)**	**1.36 (1.02–1.80)**	1.14 (0.83–1.58)	1.00	0.89 (0.64–1.25)	0.92 (0.62–1.36)	1.00	**1.49 (1.13–1.98)**	**1.41 (1.09–1.84)**	0.68 (0.38–1.23)	1.11 (0.82–1.50)	–	–	–	–	1.00	**1.39 (1.14–1.70)**
e) I find it difficult to get an appointment with a doctor at a convenient time	% (*n*)	41.5 (807)	44.1 (471)	38.4 (336)	52.8 (75)	49.0 (271)	44.3 (137)	38.7 (130)	33.0 (111)	30.9 (83)	42.1 (220)	44.9 (155)	47.7 (207)	40.3 (29)	34.3 (196)	41.3 (735)	43.0 (71)	42.2 (381)	40.8 (426)	43.0 (470)	39.7 (337)
	OR (95% CI)	–	1.00	**0.80 (0.67–0.97)**	**1.79 (1.21–2.66)**	**1.52 (1.15–2.00)**	1.27 (0.93–1.74)	1.00	0.80 (0.58–1.10)	**0.71 (0.50–0.99)**	–	–	–	–	–	–	–	–	–	–	–
f) I would be too busy to make time to go to the doctor	% (*n*)	13.6 (265)	13.1 (140)	14.3 (125)	20.4 (29)	21.0 (116)	15.9 (49)	14.6 (49)	5.7 (19)	1.1 (3)	19.9 (104)	15.4 (53)	18.4 (80)	12.5 (9)	3.3 (19)	13.5 (240)	15.2 (25)	12.1 (109)	15.0 (156)	17.0 (186)	9.3 (79)
	OR (95% CI)	–	–	–	**2.00 (1.14–3.49)**	**1.54 (1.06–2.22)**	1.09 (0.71–1.69)	1.00	**0.49 (0.28–0.86)**	**0.16 (0.04–0.54)**	1.00	0.75 (0.52–1.09)	0.87 (0.63–1.21)	**0.36 (0.16–0.82)**	**0.29 (0.17–0.50)**	–	–	–	–	–	–
g) I have too many other things to worry about	% (*n*)	13.6 (265)	14.6 (156)	12.4 (109)	19.7 (28)	19.0 (105)	17.2 (53)	11.6 (39)	7.1 (24)	6.0 (16)	16.3 (85)	15.1 (52)	15.7 (68)	16.7 (12)	8.4 (48)	13.6 (242)	13.9 (23)	14.1 (127)	13.2 (138)	15.0 (164)	11.9 (101)
	OR (95% CI)	–	–	–	**1.87 (1.10–3.18)**	**1.78 (1.20–2.65)**	**1.58 (1.01–2.46)**	1.00	**0.59 (0.34–1.00)**	**0.48 (0.26–0.88)**	–	–	–	–	–	–	–	–	–	–	–
h) I would be worried about what they might find wrong with me	% (*n*)	26.2 (509)	29.0 (310)	22.7 (199)	43.0 (61)	28.2 (156)	27.2 (84)	22.3 (75)	21.1 (71)	23.1 (62)	23.3 (122)	23.8 (82)	32.3 (140)	40.3 (29)	23.8 (136)	26.4 (470)	23.6 (39)	28.9 (261)	23.8 (248)	27.2 (298)	24.8 (210)
	OR (95% CI)	–	1.00	**0.71 (0.58–0.88)**	**2.48 (1.55–3.98)**	1.37 (1.00–1.88)	1.33 (0.93–1.91)	1.00	1.00 (0.68–1.47)	1.09 (0.70–1.71)	1.00	0.98 (0.71–1.36)	**1.51 (1.13–2.01)**	1.28 (0.71–2.32)	1.10 (0.78–1.55)	–	–	–	–	–	–
i) I would be worried about what tests they might want to do	% (*n*)	18.8 (365)	21.1 (225)	16.0 (140)	29.6 (42)	20.8 (115)	24.3 (75)	14.9 (50)	13.4 (45)	14.1 (38)	14.7 (77)	21.7 (75)	22.8 (99)	26.4 (19)	16.6 (95)	18.8 (334)	18.8 (31)	20.0 (180)	17.7 (185)	19.0 (208)	18.5 (157)
	OR (95% CI)	–	1.00	**0.73 (0.58–0.93)**	**2.36 (1.40–3.98)**	**1.53 (1.06–2.21)**	**1.89 (1.27–2.83)**	1.00	0.87 (0.55–1.38)	0.87 (0.52–1.48)	1.00	**1.59 (1.11–2.28)**	**1.67 (1.20–2.34)**	1.35 (0.70–2.61)	**1.52 (1.03–2.25)**	–	–	–	–	–	–
j) I wouldn't feel confident talking about my symptom(s) with the doctor	% (*n*)	8.6 (168)	9.7 (104)	7.3 (64)	11.3 (16)	9.6 (53)	7.4 (23)	9.8 (33)	7.4 (25)	6.7 (18)	5.7 (30)	8.1 (28)	11.3 (49)	11.1 (8)	9.3 (53)	8.6 (152)	9.7 (16)	9.2 (83)	8.2 (85)	7.8 (85)	9.7 (82)
	OR (95% CI)	–	–	–	–	–	–	–	–	–	1.00	1.45 (0.85–2.48)	**2.09 (1.30–3.36)**	2.05 (0.90–4.67)	**1.68 (1.06–2.68)**	–	–	–	–	–	–
k) I have had a bad experience at the doctor's in the past	% (*n*)	16.0 (312)	18.6 (199)	12.9 (113)	15.5 (22)	22.4 (124)	14.9 (46)	17.6 (59)	8.9 (30)	11.5 (31)	16.6 (87)	18.0 (62)	19.6 (85)	13.9 (10)	11.9 (68)	16.1 (287)	15.2 (25)	14.5 (131)	17.4 (181)	13.4 (146)	19.5 (165)
	OR (95% CI)	–	1.00	**0.68 (0.53–0.88)**	1.25 (0.72–2.20)	**1.67 (1.16–2.39)**	0.92 (0.60–1.41)	1.00	**0.45 (0.28–0.72)**	**0.57 (0.35–0.92)**	–	–	–	–	–	–	–	1.00	**1.30 (1.00–1.68)**	1.00	**2.16 (1.65–2.82)**
l) I would be worried the doctor wouldn't take my symptom(s) seriously	% (*n*)	18.4 (357)	20.9 (223)	15.3 (134)	23.9 (34)	24.8 (137)	18.1 (56)	15.5 (52)	14.0 (47)	11.5 (31)	17.6 (92)	18.6 (64)	23.3 (101)	23.6 (17)	14.5 (83)	18.1 (321)	21.8 (36)	18.1 (163)	18.6 (194)	18.1 (198)	18.6 (158)
	OR (95% CI)	–	1.00	**0.71 (0.56–0.90)**	**1.95 (1.19–3.20)**	**1.97 (1.37–2.83)**	1.29 (0.85–1.95)	1.00	0.91 (0.59–1.40)	0.69 (0.42–1.11)	–	–	–	–	–	–	–	–	–	1.00	**1.34 (1.04–1.72)**
m) I don't want to be seen as somebody who makes a fuss	% (*n*)	34.8 (676)	36.7 (392)	32.4 (284)	34.5 (49)	33.6 (186)	33.3 (103)	33.6 (113)	35.4 (119)	39.4 (106)	32.1 (168)	33.9 (117)	33.9 (147)	43.1 (31)	37.3 (213)	35.6 (632)	26.7 (44)	37.4 (337)	32.5 (339)	32.8 (359)	37.4 (317)
	OR (95% CI)	–	–	–	–	–	–	–	–	–	–	–	–	–	–	1.00	**0.64 (0.45–0.92)**	1.00	**0.80 (0.66–0.96)**	–	–
n) I don't like having to talk to the GP receptionist about my symptom(s)	% (*n*)	39.5 (769)	43.2 (462)	35.1 (307)	34.5 (49)	43.6 (241)	37.5 (116)	39.9 (134)	41.4 (139)	33.5 (90)	35.0 (183)	43.2 (149)	43.3 (188)	44.4 (32)	38.0 (217)	40.2 (715)	32.1 (53)	37.6 (339)	41.2 (430)	38.6 (422)	40.9 (347)
	OR (95% CI)	–	1.00	**0.72 (0.59–0.86)**	0.66 (0.41–1.05)	1.22 (0.92–1.62)	0.93 (0.68–1.29)	1.00	1.10 (0.79–1.52)	0.76 (0.51–1.12)	1.00	**1.39 (1.05–1.85)**	**1.46 (1.12–1.90)**	**2.10 (1.17–3.78)**	1.24 (0.92–1.68)	1.00	**0.64 (0.45–0.91)**	–	–	–	–

Bold indicates a significant odds ratio (P < 0.05).

a Composition: 14.1: never worked *n* = 68/14.2: long-term unemployed *n* = 20/16.0; occupations not classified or inadequately stated *n* = 0/17.0; and not classifiable for other reasons *n* = 497.

b Multivariate backwards stepwise logistic regression. All ORs are adjusted for age, gender, socio-economic status, ethnic group, relationship status and long-term illness. ‘Do not know’ responses and refusals treated as missing.

## Appendix A Materials and methods

### Survey design and sampling

The ONS survey recruits using random probability sampling stratified by region, the proportion of households with no car, the proportion of households classified as NS-SEC categories 1–3 (managerial, professional and intermediate occupations) and the proportion of people aged over 65 years. Households are randomly selected from the Royal Mail's Postcode Address File of ‘small users’ and individuals from each household are selected using a Kish grid.

### Item wording

A. Which of the following might put you off going to the doctor? Please choose your answer from this card:

• Strongly agree
• Agree
• Neither agree nor disagree
• Disagree
• Strongly disagree

Random order

MBR_3a: I find it embarrassing talking to the doctor about my symptoms.

MBR_3b: I would be worried about wasting the doctor's time.

MBR_3c: My doctor is difficult to talk to.

MBR_3d: I find it difficult to get an appointment with a particular doctor.

MBR_3e: I find it difficult to get an appointment with a doctor at a convenient time.

MBR_3f: I would be too busy to make time to go to the doctor.

MBR_ 3g: I have too many other things to worry about.

MBR_3h: I would be worried about what they might find wrong with me.

MBR_3i: I would be worried about what tests they might want to do.

MBR_3j: I wouldn't feel confident talking about my symptom(s) with the doctor.

MBR_3k: I have had a bad experience at the doctor's in the past.

MBR_3l: I would be worried the doctor wouldn't take my symptom(s) seriously.

MBR_3m: I don't want to be seen as somebody who makes a fuss.

MBR_3n: I don't like having to talk to the GP receptionist about my symptom(s).

B. Any long-standing illness, disability or infirmary?

Yes.

No.

### Demographic variables:

• Age (agex): 16–24, 25–44, 45–54, 55–64, 65–74 and 75+
• Sex (rsex): Male and Female
• Relationship status (DeFact): married/civil partnership/cohabiting and single/widowed/divorced/former civil-partner/separated
• Ethnicity (Ethnici01): White and other ethnic backgrounds
• Occupation (nsecac3): managerial/professional, intermediate/small employers/lower supervisory, semi-routine/routine, full-time students and non-workers (those who have been out of work for longer than eight years, are long-term unemployed or have never worked or are unclassifiable for another reason).
